# *Leucocytozoon* infections in tits (Aves, Paridae): blood and tissue stages investigated using an integrative approach

**DOI:** 10.1051/parasite/2025007

**Published:** 2025-02-21

**Authors:** Germán Alfredo Gutiérrez-Liberato, Mélanie Duc, Vytautas Eigirdas, Carolina Romeiro Fernandes Chagas

**Affiliations:** 1 Nature Research Centre Akademijos g. 2 08412 Vilnius Lithuania; 2 Tadas Ivanauskas Zoological Museum, Ventės Ragas Ornithological Station Marių 24 Kintai 99361 Lithuania

**Keywords:** Haemosporida, Passeriformes, Cytochrome *b* gene, Chromogenic *in situ* hybridization, Microscopical analysis, Molecular techniques

## Abstract

*Leucocytozoon* species are cosmopolitan and prevalent avian parasites, with some infections being lethal, mainly due to the exo-erythrocytic development of the parasite in bird tissues. The patterns of exo-erythrocytic development in *Leucocytozoon* spp. infections in wild birds remain poorly studied. This study investigated the development of *Leucocytozoon* spp. tissue stages in tits (Paridae). Great tits (*Parus major*), Blue tits (*Cyanistes caeruleus*), and Coal tits (*Periparus ater*) were screened for infections using an integrative approach that consisted of microscopic analysis of thin blood smears, histological techniques, chromogenic *in situ* hybridization (CISH), PCR-based methods, and phylogenetic analysis. In total, 41 individuals were analyzed (eight naturally infected that were selected and euthanized, and 33 found dead in the wild and opportunistically sampled). Among the naturally infected birds, all individuals that were microscopically positive for *Leucocytozoon* species were also PCR-positive for these parasites. Co-infections with *Plasmodium* spp. and *Haemoproteus* spp. were commonly found, mainly among the opportunistically sampled birds. Two morphotypes were identified, *Leucocytozoon majoris* (Laveran, 1902) and *Leucocytozoon fringillinarum* Woodcock, 1910. Tissue stages were present in three birds sampled exclusively during the non-breeding season, two of them with meronts developing in the kidneys and liver, and one individual with a megalomeront in the heart. All the exo-erythrocytic stages were confirmed to be *Leucocytozoon* spp. by CISH using a *Leucocytozoon* genus-specific probe. Phylogenetic analysis placed parasite lineages with different morphotypes in separate clades. The developmental patterns of exo-erythrocytic stages of *Leucocytozoon* spp. in naturally infected passerines are poorly understood, requiring further research.

## Abstract (fr)

Les espèces de *Leucocytozoon* sont des parasites aviaires cosmopolites et répandus. Certaines infections sont mortelles, principalement en raison du développement exo-érythrocytaire du parasite dans les tissus des oiseaux. Les schémas de développement exo-érythrocytaire dans les infections à *Leucocytozoon* spp. chez les oiseaux sauvages restent peu étudiés. Cette étude a examiné le développement des stades tissulaires de *Leucocytozoon* spp. chez les mésanges (Paridae). Des mésanges charbonnières (*Parus major*), mésanges bleues (*Cyanistes caeruleus*) et mésanges noires (*Periparus ater*) ont été examinées pour détecter les infections à l’aide d’une approche intégrative consistant en une analyse microscopique de frottis sanguins minces, des techniques histologiques, une hybridation chromogène *in situ* (CISH), des méthodes basées sur la PCR et une analyse phylogénétique. Au total, 41 individus ont été analysés (huit naturellement infectés qui ont été sélectionnés et euthanasiés et 33 trouvés morts dans la nature et échantillonnés de manière opportuniste). Parmi les oiseaux naturellement infectés, tous les individus qui étaient microscopiquement positifs pour les espèces de *Leucocytozoon* étaient également positifs pour ces parasites par PCR. Des co-infections avec *Plasmodium* spp. et *Haemoproteus* spp. ont été couramment trouvées, principalement parmi les oiseaux échantillonnés de manière opportuniste. Deux morphotypes ont été identifiés, *Leucocytozoon majoris* (Laveran, 1902) et *Leucocytozoon fringillinarum* Woodcock, 1910. Des stades tissulaires étaient présents chez trois oiseaux échantillonnés exclusivement en dehors de la saison de reproduction, deux d’entre eux avec des mérontes se développant dans les reins et le foie, et un individu avec un mégaloméronte dans le cœur. Tous les stades exo-érythrocytaires ont été confirmés comme étant des *Leucocytozoon* spp. par CISH en utilisant une sonde spécifique au genre *Leucocytozoon*. L’analyse phylogénétique a placé les lignées de parasites avec différents morphotypes dans des clades distincts. Les modèles de développement des stades exo-érythrocytaires de *Leucocytozoon* spp. chez les passereaux naturellement infectés sont mal compris et nécessitent des recherches supplémentaires.

## Introduction

Parasites from the order Haemosporida comprise a wide range of species that infect several vertebrate hosts. These vector-borne blood parasites are of medical and veterinary importance as etiological agents of human malaria, and several other diseases in poultry and wild birds [1, 6, 10, 14, 37, 43, 49, 55]. Some parasites, such as *Plasmodium* spp., can be found in a high diversity of vertebrate hosts, while other haemosporidians are found exclusively in birds, which is the case for those belonging to the genus *Leucocytozoon* [49].

Haemosporidian parasites have a worldwide distribution, and infections have been reported from almost all zoogeographic regions, except Antarctica [50]. Currently, there are approximately 50 species of *Leucocytozoon*, which were described based mainly on a combination of their morphological features, ecological traits, and development patterns in their vertebrate hosts [48–50]. However, there is a marked discrepancy between the number of morphospecies and available molecular information, with more than 1,500 cytochrome *b* (*cytb*) gene lineages reported in MalAvi (MalAvi database, http://130.235.244.92/Malavi/ accessed on February 22, 2024) [4] and only a few of them (24) are linked to a morphospecies [4, 50], with 12.2% (3) of them reported in Paridae birds, also known as tits and chickadees [4].

Morphological description of blood stages of *Leucocytozoon* spp. is complicated even for experienced parasitologists [50]. Their unique morphological features (absence of hemozoin pigments, no merogony in the peripherical blood, and the conspicuous deformation on the infected host cell) form a parasite-host cell complex, making them easy to differentiate from other closely related parasites, such as those from the genera *Haemoproteus* and *Plasmodium* [32, 49], but not within the *Leucocytozoon* genus. Due to the lack of structures, the identification and differentiation of *Leucocytozoon* species has mainly been done relating to the bird order and the influence of the parasites on the infected host cell [49]. For instance, nine *Leucocytozoon* morphospecies have been described to infect Passeriformes: five with only roundish parasite-host cell complex (*Leucocytozoon majoris, L. sakharoffi, L. berestneffi, L. fringillinarum,* and *L. dubreuili*), two with only fusiform parasite-host cell complex (*L. grallarie* and *L. neotropicalis*), and two with both parasite-host cell complexes (*L. balmorali* and *L. maccluri*) [50]. The most common parasite-host cell complex found in passerine birds is the roundish one, but some species are found only in birds belonging to the Corvidae family, such as *L. sakharoffi* and *L. berestneffi* [49]. The other *Leucocytozoon* spp. forming roundish parasite-host cell complexes are differentiated based on the shape assumed by the nucleus of the infected cell. A host cell nucleus with a band-like shape is characteristic of *L. majoris* (Laveran, 1902), a dumbbell-like shape of *L. dubreuili*, and a cap-like shape of *L. fringillinarum* Woodcock, 1910 [49].

Concerning knowledge about the exo-erythrocytic stages, the information available for *Leucocytozoon* spp. is scarce and fragmentary, especially in Passeriformes [50]. Research on tissue stages development has focused on *L. caulleryi* infecting chickens [10, 36, 37], *L. smithi* infecting turkeys [55], and *L. simondi* infecting ducks [26], mainly due to their economic importance, with a few studies in wild birds [2, 9, 27, 30]. Investigations of the tissue development of *Leucocytozoon* sp. were mainly done by experimental infection several decades ago [15, 18, 49, 51, 56] and currently, few studies have focused on studying the development of these stages in natural infections in wild birds [9, 24, 27]. Additionally, the microscopic study of histological sections is a time-consuming methodology that requires expertise to correctly recognize a parasite stage from other possible structures with which it could be confused, such as cellular debris or tissue necrosis [24]. The application of chromogenic *in situ* hybridization (CISH) has allowed a more accurate diagnosis of different haemosporidians in microscopic analyses of histological sections [9, 16, 17, 24, 25, 27].

The Paridae birds comprise a diverse group of small passerine birds with 13 genera and 63 species and are known for a high prevalence of *Leucocytozoon* spp. infections [33], as well as co-infections with other closely related haemosporidian parasites [38]. With that in mind, this study aimed (i) to investigate exo-erythrocytic development of *Leucocytozoon* spp. in Paridae birds; (ii) to identify genetic lineages involved in these infections and link them with the different *Leucocytozoon* sp. morphotypes; and (iii) to check the phylogenetic relationships between *Leucocytozoon* spp. lineages infecting Paridae birds.

## Materials and methods

### Ethics

Specimens were collected under collection permits approved by the Lithuania Environmental Protection Agency, Vilnius (2020 04 08 Nr. A4E-2892; 2021 05 05 Nr. SR-96, and 2023 03 15 Nr. SR-197).

### Study site and sampling

Individuals belonging to the Paridae family were sampled at the Ventės Ragas ornithological station (55°20′38.93″ N, 21°11′34.05″ E), Lithuania during the spring (May) 2021, autumn (September and October) seasons of 2020 and 2021, and autumn (October) 2023. The birds were caught using a large stationary trap and mist nets. Blood samples were obtained from the brachial vein by puncturing with a needle. A few drops of blood were used to prepare thin blood smears from each individual, and the remaining blood was stored in SET-buffer (0.05 M tris, 0.15 M NaCl, 0.5 M EDTA, pH 8.0) for subsequent molecular analyses. The thin blood smears were dried at room temperature with a battery-operated fan and fixed with absolute methanol (~1 s). One slide per individual was stained with a 30% Giemsa solution for 15 min [7]. Microscopic examination was immediately conducted to detect the presence of *Leucocytozoon* sp. gametocytes in the field, following the available protocols [52]. The remaining slides were stained with 10% Giemsa solution for 1 h [52], examined for at least 30 min to identify the parasite morphotype, and to calculate the parasitemia. The intensity of parasitemia was determined as a percentage of parasites per 10,000 red blood cells [20]. Representative preparations of blood stages (49759NS–49764NS) were deposited at the Nature Research Centre, Vilnius, Lithuania.

Eight infected individuals were selected and euthanized by decapitation according to available permits (see Ethics). The internal organs were collected and stored for histological examination (described below). Furthermore, 33 individuals that were found dead at the study site and in its surrounding areas, during the autumn season (September, October, and November) 2022, and frozen until dissections in May 2023, were included in this study (Table 1). Blood cloths were collected from the heart and stored in SET-buffer for molecular analyses, and internal organs were processed as for the other birds. No blood smears were prepared for these birds.

**Table 1:** Examined Paridae birds, with the results on thin blood smears microscopy, parasite cytochrome *b* gene (*cytb*) lineages, and histology using hematoxylin-eosin (H&E) staining and chromogenic *in situ* hybridization (CISH).

Common name (host species)	Individual	Season (month)	Age	Sex	Microscopy (*Leucocytozoon* parasitemia)	Parasite *cytb* lineages	H&E (infected organ)	CISH (infected organ)
Eurasian Blue tit (*Cyanistes caeruleus*)	H10/20R	A (September)	AD	M	L (0.05%)^a^	lPARUS4 (*Leucocytozoon* sp.)	–	BS
	H21/20R	A (September)	JUV	M	L (0.06%)^a^	lSYBOR07 (*Leucocytozoon* sp.)	–	BS
	H70/21R	A (October)	U	U	NP	Co-infection (*Leucocytozoon* spp.)	–	BS
	H9/23R	A (October)	U	U	NP	lPARUS4 (*Leucocytozoon* sp.)	–	BS
	H10/23R	A (October)	U	M	NP	lPARUS14 (*Leucocytozoon* sp.)	–	–
	H17/23R	A (October)	U	U	NP	–	–	–
	H18/23R	A (October)	U	U	NP	–	–	–
	H24/23R	A (October)	AD	F	NP	pTURDUS1 (*Plasmodium circumflexum*)	–	–
	H30/23R	A (October)	U	U	NP	pTURDUS1 (*P. circumflexum*)	–	–
	H32/23R	A (October)	U	U	NP	pTURDUS1 (*P. circumflexum*)	–	–
	H34/23R	A (October)	U	U	NP	lPARUS4 (*Leucocytozoon* sp.)	–	–
	H35/23R	A (October)	U	U	NP	–	–	–
	H36/23R	A (October)	U	U	NP	pTURDUS1 (*P. circumflexum*)	–	–
	H38/23R	A (November)	AD	M	NP	lPARUS14 (*Leucocytozoon* sp.)	–	–
	H39/23R	A (October)	AD	F	NP	lPARUS95* (*Leucocytozoon* sp.)	–	–
	H48/23R	U	U	U	NP	pTURDUS1, *(P. circumflexum)*	–	–
						Co-infection (*Leucocytozoon* spp.)		
	H49/23R	U	U	U	NP	pBT7 (*Plasmodium* sp.)	–	–
	H50/23R	U	U	U	NP	–	–	–
	H127/23R	A (September)	U	F	NP	–	–	–
	H135/23R	A (October)	U	U	NP	–	–	–
Great tit (*Parus major*)	H44/21R	S (May)	U	U	L (0.03%)^a^	Co-infection (*Leucocytozoon* spp.)	M (kidneys)	BS, M (kidneys)
	H56/21R	A (October)	U	U	H¨, L (0.004%)^a^	lPARUS16 (*Leucocytozoon* sp.)	–	BS
	H58/21R	A (October)	U	M	L (0.09%)^a^	lPARUS16 (*Leucocytozoon* sp.)	M (kidneys, liver)	BS, M (liver)
	H81/21R	A (October)	U	M	H, L (0.002%)^a^	hPARUS1 (*Haemoproteus majoris*)	–	BS
						lPARUS19 (*Leucocytozoon* sp.)		
	H83/21R	A (October)	AD	F	L (0.01%)^b^	lPARUS22 (*Leucocytozoon* sp.)	MM (heart)	BS, MM (heart)
	H84/21R	A (October)	AD	F	P, L (0.004%)^b^	pTURDUS1 (*P. circumflexum*)	–	BS
						lPARUS22 (*Leucocytozoon* sp.)		
	H14/23R	A (October)	U	U	NP	pTURDUS1 (*P. circumflexum*)	–	–
	H15/23R	A (October)	U	U	NP	pTURDUS1 (*P. circumflexum*)	–	–
						Co-infection (*Leucocytozoon* spp.)		
	H16/23R	A (October)	U	U	NP	pTURDUS1 (*P. circumflexum*)	–	–
						lPARUS4 (*Leucocytozoon* sp.)		
	H20/23R	A (October)	AD	M	NP	pBT7 (*Plasmodium* sp.)	–	–
	H23/23R	A (September)	U	U	NP	pTURDUS1 (*P. circumflexum*)	–	–
						lPARUS25 (*Leucocytozoon* sp.)		
	H31/23R	A (October)	U	U	NP	–	–	–
	H33/23R	A (October)	U	U	NP	lPARUS19 (*Leucocytozoon* sp.)	–	–
	H123/23R	A (October)	U	M	NP	lPARUS4 (*Leucocytozoon* sp.)	–	BS
	H130/23R	A (October)	U	U	NP	lPARUS4 (*Leucocytozoon* sp.)	–	BS
	H131/23R	A (October)	U	U	NP	–	–	–
	H132/23R	A (October)	U	U	NP	–	–	–
	H133/23R	A (October)	U	U	NP	–	–	–
Coal tit (*Periparus ater*)	H12/23R	A (October)	U	M	NP	–	–	–
	H13/23R	A (October)	U	U	NP	–	–	–
	H29/23R	A (October)	U	U	NP	pBT7 (*Plasmodium* sp.)	–	–

A: Autumn, S: Spring, AD: adult. JUV: juvenile. M: male. F: female. U: Unknown. NP: No Preparation as bird was retrieved dead. L: *Leucocytozoon*. H: *Haemoproteus*. P: *Plasmodium*. (^a^): *L. majoris* morphotype. (^b^): *L. fringillinarum* morphotype. BS: Blood Signal. (–): negative result. (*): new lineage. M: meront. MM: megalomeront. Note: H¨: *Haemoproteus* lineage in sample H56/21R could not be amplified.

### DNA extraction, PCR, and sequencing

DNA was extracted from the blood samples stored in SET-buffer using an ammonium acetate protocol [40]. A standard nested PCR protocol was performed targeting a ~ 480 bp fragment of the parasite *cytb* gene. The set of primer HaemNFI/HaemNR3 was used in the first run, and the second PCR was done with two sets of primers: HaemFL/HaemR2L which amplifies *Leucocytozoon* spp. DNA, and HaemF/HaemR2 which amplifies *Haemoproteus* spp. and *Plasmodium* spp. DNA. The thermal cycle profile was the same as described in the original protocols [23, 54]. On each PCR reaction, microscopically positive samples for *Leucocytozoon* spp.*, Haemoproteus* spp.*/Plasmodium* spp. and ultrapure water were used as positive and negative controls, respectively. PCR products were visualized in a 2% agarose gel and positive amplifications were purified using differential precipitation with ammonium acetate protocol [40]. Positive samples were sequenced from both ends with the respective primers, using Big Dye Terminator V3.1 Cycle Sequencing Kit and ABI PRISMTM 3100 capillary sequencing robot (Applied Biosystems, Foster City, CA, USA). The resulting sequences were analyzed using Geneious Prime 2023.2.1 (https://www.geneious.com). Both strands were aligned to form a contig sequence and analyzed for the presence of double peaks and quality. Co-infection with other lineages was considered if double peaks were found. These sequences were not included in the phylogenetic analysis.

Obtained lineages were compared with other sequences deposited in the MalAvi [4] and GenBank (https://www.ncbi.nlm.nih.gov/ accessed on February 22, 2024) databases using BLAST. Lineage identities were considered when the obtained contig had 100% similarity with existing lineages deposited in the MalAvi database. If the obtained sequences had at least one base pair of difference, they were considered new lineages and named according to the MalAvi protocol [4]. All obtained sequences were deposited in both the GenBank (PP988073–PP988098) and MalAvi databases.

### Phylogenetic analysis

A total of 92 *Leucocytozoon* spp. lineage sequences reported in the MalAvi database from Paridae birds, as well as the ones linked to a morphospecies, were used for the phylogenetic analysis (see https://www.parasite-journal.org/10.1051/parasite/2025007/olm). The alignment was constructed using MEGA 11 V11.0.13 [47]. A Bayesian Inference (BI) analysis was carried out in CIPRES Science Gateway V3.3 [35]. Sequences from other haemosporidian parasites (*Leucocytozoon* (*Akiba*) sp., and *Haemoproteus* sp.) were used as outgroups. This analysis was performed under the general time-reversal model (GTR + I + G) suggested by jModelTest 2.1.1 [12]. For the BI, independent Markov Chain Monte Carlo (MCMC) simulations were run simultaneously with six chains; using 1 × 10^7^ generations sampled every 500 generations. After discarding 25% of the trees as “burn-in”, the remaining trees were used to build the majority rule consensus tree, which was visualized and edited using FigTree version 1.4.3 [39] and MEGA 11 V11.0.13 [47]. Genetic distances within and between groups were estimated using a Kimura two-parameter model of substitution, implemented in MEGA 11 V11.0.13 [47].

### Histological examination and chromogenic *in situ* hybridization (CISH)

The organs collected (brain, heart, trachea, lungs, kidneys, liver, spleen, pancreas, esophagus, proventriculus, gizzard, intestine, reproductive organs, pectoral, and leg muscles) were fixed in 10% neutral formalin for 24 h, then washed in distilled water for 1 h, and stored in ethanol 70% until processing [46]. Later, tissue samples were dehydrated with increasing ethanol concentrations (from 70 to 100%), clarified in xylene, and embedded in paraffin wax-blocks [46]. Histological sections of 3 μm were prepared with a microtome from all the paraffin blocks containing the collected material. At least four sections per organ were prepared and stained with hematoxylin and eosin-staining procedures, hereafter H&E, and one section per organ was used for application of the CISH method. Histological preparations were mounted on glass slides for H&E and on Fisherbrand™ Superfrost™ Plus Microscope Slides (Thermo Fisher Scientific, Waltham, MA, USA) for CISH. The latter was applied using the genus-specific oligonucleotide probe (Leuco*18S*), following the original protocol [24].

H&E preparations were used to investigate exo-erythrocytic stages of *Leucocytozoon* spp. using 100x, 200x, and 400x magnifications with an Olympus CX23 or BX51 microscope (Olympus, Tokyo, Japan). Positive preparations were photographed using a DP12 camera with the software DP-SOFT (Olympus) [24]. Representative preparations (49765NS–49772NS) were deposited at the Nature Research Centre, Vilnius, Lithuania.

## Results

### Microscopy findings

**Figure 1:**
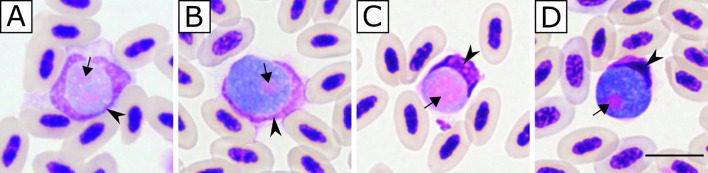
Gametocytes of *Leucocytozoon* spp. with roundish parasite-host cell complexes from Great tit *Parus major* blood samples. Two morphotypes were found during microscopic examination: *Leucocytozoon majoris* (A, B) in the individual H10/20R (lPARUS4) and *Leucocytozoon fringillinarum* (C, D) in the individual H83/21R (lPARUS22). Microgametocytes (A, C). Macrogametocytes (B, D). Short simple black arrow: parasite nucleus. Black arrowhead: nucleus of infected host cell. Methanol-fixed and Giemsa-stained. Scale bar 10 μm.

In total, blood samples from eight individuals, two Eurasian Blue tits *Cyanistes caeruleus* and six Great tits *Parus major*, were microscopically positive for *Leucocytozoon* spp. (Table 1). Microscopic analyses of thin blood smears revealed the presence of two *Leucocytozoon* sp. morphotypes: *L. majoris* (present in six individuals, two Blue tits and four Great tits; Table 1, Figs. 1A–1B) and *L. fringillinarum* (present in two Great tits; Table 1 and Figs. 1C–1D). Parasitemia intensity for the *L. majoris* morphotype ranged from 0.002% to 0.09% and for the *L. fringillinarum* morphotype from 0.004% to 0.01% (Table 1). Co-infections with other haemosporidian parasites of the genera *Plasmodium* and *Haemoproteus* were also detected in three individuals, all Great tits (Table 1). For the individuals that were found dead, parasitemia was not determined since clotted blood is not suitable for the preparation of thin blood smears and microscopic analysis (Table 1).

### Parasite molecular diversity and phylogenetic analysis

Using PCR methods, *Leucocytozoon* spp. infections were confirmed in all the samples that were positive by microscopy. Single *Leucocytozoon* sp. infections were confirmed in five individuals and with four different lineages (Table 1). One individual had co-infection with different *Leucocytozoon* spp. lineages (individual H44/21R); one individual with *Plasmodium circumflexum (cytb* lineage pTURDUS1) (individual H84/21R), and one individual with *Haemoproteus majoris* (hPARUS1) (individual H81/21R).

Among the opportunistically sampled individuals, PCR-based methods detected single infections by *Leucocytozoon* sp. in eight individuals, and three individuals had co-infection with different *Leucocytozoon* spp. lineages (Table 1). Four individuals had co-infection with *P. circumflexum* pTURDUS1. No co-infections with *Haemoproteus* sp. were detected by molecular methods. Single infections with *Plasmodium* sp. were detected in eight individuals, three with *Plasmodium* sp. pBT7 and five with *P. circumflexum* pTURDUS1 (Table 1).

Seven of the eight *Leucocytozoon* spp. lineages recovered in the analyzed birds were previously reported in Paridae birds, and one new lineage was found in a Great tit (*Leucocytozoon* sp. lPARUS95). The most prevalent lineage in this study was *Leucocytozoon* sp. lPARUS4 (Table 1). Furthermore, the prevalence of infection with *P. circumflexum* pTURDUS1 was high, being reported in ten individuals.

**Figure 2:**
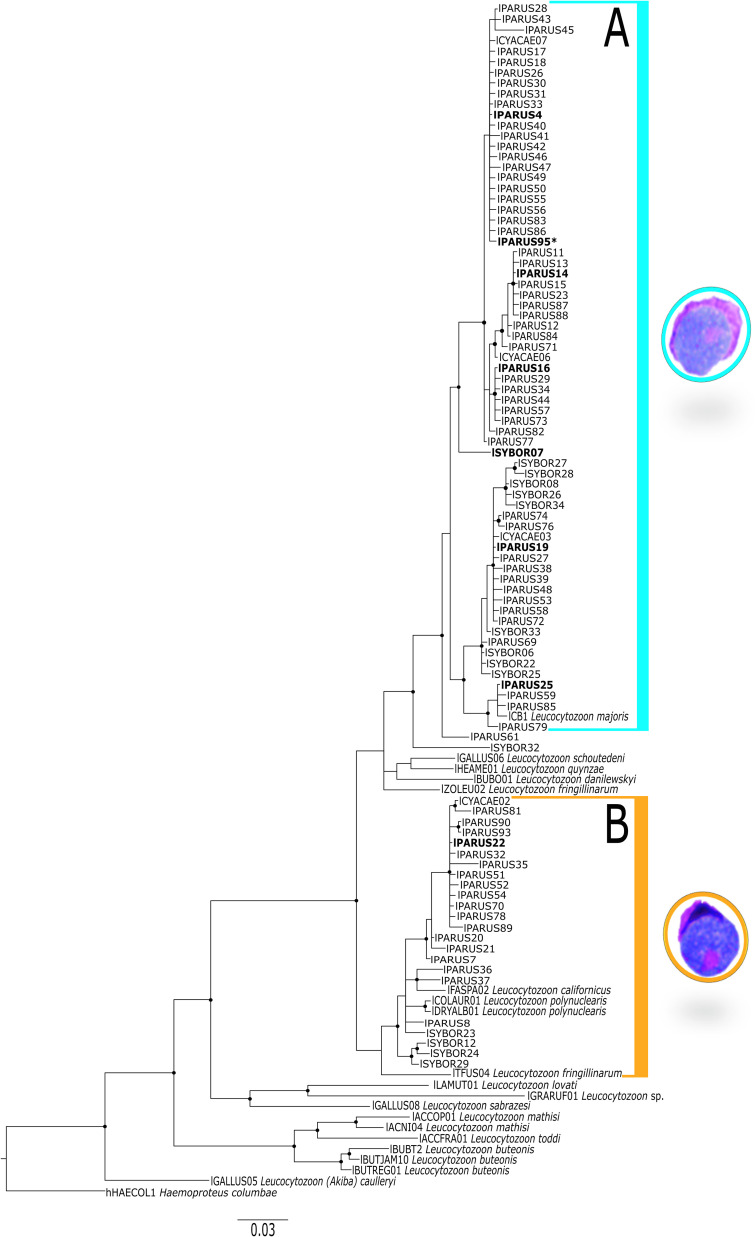
Bayesian inference tree of partial cytochrome *b* gene sequences of 92 *Leucocytozoon* lineages and sequences linked to a morphospecies. Two lineages (*Haemoproteus columbae* hHAECOL01 and *Leucocytozoon (Akiba) caullery* lGALLUS05) were used as the outgroup. Parasite lineages were presented as they appear in the MalAvi database. Two clades were highlighted according to the morphotypes some of the lineages were linked to, being *L. majoris* (A) and *L. fringillinarum* (B). Bold font indicates the lineages obtained in this study. The new lineage is indicated with an asterisk. All the posterior probabilities ≥ 0.7 were provided with a black dot. GenBank accession numbers of the sequences used in the phylogeny are given in Supplementary Table S1.

In the phylogenetic analysis, all *Leucocytozoon* lineages that presented *L. majoris* morphotype (lPARUS4, lSYBOR07, lPARUS16 and lPARUS19) were placed in the same clade, together with the other lineages obtained from the opportunistically sampled birds (lPARUS14, lPARUS95, and lPARUS25) (Fig. 2A). The estimated genetic divergence within clade A was 0.027. Both samples that presented gametocytes with the *L. fringillinarum* morphotype had the same parasite lineage *Leucocytozoon* sp. lPARUS22 that was placed in a separate clade (Fig. 2B). The estimated genetic divergence within clade B was 0.030. Additionally, the estimated genetic divergence between clades A and B was 0.079.

### Histological findings

**Figure 3:**
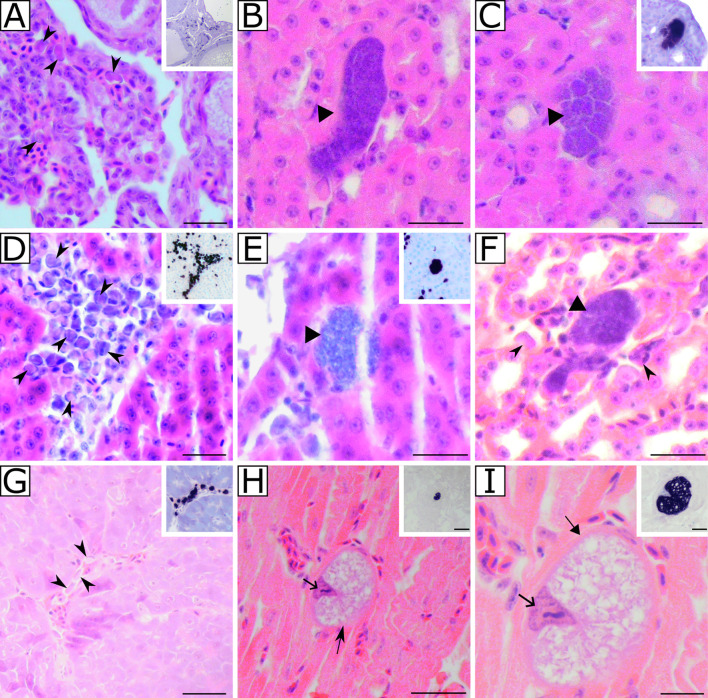
*Leucocytozoon* spp. exo-erythrocytic and blood stages in naturally infected Great tits *Parus major*. Individual H44/21R with *Leucocytozoon* spp. (molecular co-infection) (A–C) presented blood stages in oviduct (A) and meront development found in the kidneys (B, C). Tissue stages visualized in H&E-stained preparations were confirmed by chromogenic *in situ* hybridization (CISH) using a *Leucocytozoon* genus-specific probe (Leuco*18S*) indicated by a purple signal in the inserts of panels. Individual H58/21R infected with *Leucocytozoon* sp. (cytochrome *b* lineage lPARUS16) (D–F) had blood stages in the liver vessels (D) as well as meronts in the liver (E) and kidneys (F). All meronts seemed to be growing, as merozoites were not recognizable. Individual H83/21R infected with *Leucocytozoon* sp. (lPARUS22) (G–I) had blood stages in the spleen (G) and a megalomeront developing in the heart (H, I). The megalomeront, covered by a thick eosinophilic wall, showed growing cytomeres. Black arrowheads: gametocytes. Black triangle arrowheads: meronts. Wide black arrow: enlarged host cell nucleus. Simple black arrow: megalomeront-wall. Scale bars: A, D, G, I 20 μm. B–C, E–F, H 50 μm. (insert I, 50 μm. All remaining inserts, 20 μm).

*Leucocytozoon* spp. meronts were seen in the histological preparations stained with H&E of two Great tits (Figs. 3B, 3C, 3E, 3F), and one megalomeront was observed in a third individual (Table 1, Figs. 3H, 3I). No tissue stages were found in the Eurasian Blue tits or in the Coat tits. A high number of blood stages (present in the H&E preparations and CISH-treated samples) were found in different organs, including the oviducts (Figs. 3A, 3D). Blood stages were not evident in the brain, pectoral muscle, and leg muscle.

Two meronts were found in the individual H44/21R (undetermined lineage) in the kidneys (Figs. 3B–3C), one presenting an elongated shape and darker coloration (note the strong purple staining of the structure) of approximately 50 μm in length and no evidence of cytomere formation, a feature of early growing meronts (Fig. 3B); while the other one had an oval shape, ≤40 μm in width and marked segmentation that is associated with cytomere formation (Fig. 3C). These results were confirmed by CISH-treated histological sections (Fig. 3 inserts). No signs of necrosis or inflammation were found near these meronts.

In the individual H58/21R (lPARUS16), the meronts were found in the liver (Fig. 3E) and kidneys (Fig. 3F). At least five meronts were seen in the liver, and four in the kidneys. These meronts had differences in length and shape, depending on the organ they were infecting. In the kidneys, the meronts were irregularly shaped, without cytomere differentiation, length not exceeding 50 μm, and present mainly in the spaces of the renal tubules (Fig. 3F). In the liver, developing meronts were seen presenting round forms, and with lengths no longer than 30 μm, they were mainly seen in the hepatic sinusoids (Fig. 3E). Of note, a high number of blood stages was seen in the liver; these could be evidenced both in the H&E slides and in the histological preparations treated with CISH (Fig. 3D). No signs of necrosis or inflammation were seen in any of the organs of this individual. Additionally, the brain was the only organ where blood stages were not seen.

A megalomeront was found in the heart of the individual H83/21R (lPARUS22) (Figs. 3H, 3I). The length of this structure was approximately 60 μm width, and it was covered by a conspicuous capsule-shaped wall (Fig. 3I). The large number of unstained areas that look like empty spaces and the strong signal obtained with the Leuco18S probe during CISH suggest that it is a growing stage (Fig. 3I). No other tissue stages were seen in this individual. However, blood stages were observed in histological sections of almost all organs (Fig. 3G). No signs of inflammation or necrosis were observed.

## Discussion

The presence of *Leucocytozoon* spp. exo-erythrocytic development in naturally infected Great tits was confirmed in this study. Meronts were seen in the liver and kidneys, and a megalomeront in the cardiac muscle (Fig. 3). The presence of co-infections was common in the studied individuals (eight out of 29 PCR-positive individuals; Table 1). Morphological identification of *Leucocytozoon* species was not possible; however, parasites that resemble two *Leucocytozoon* sp. morphotypes were found, *L. majoris* and *L. fringillinarum* (Fig. 1, Table 1). Phylogenetic analysis placed all the lineages found in samples presenting *L. majoris* morphotype in the same clade and separated from the one with *L. fringillinarum* morphotype (Fig. 2).

The presence of co-infections with other haemosporidian parasites (*Haemoproteus* sp. and *Plasmodium* sp.) was evident by microscopy. Except one, all other microscopically detected co-infections were confirmed by PCR (Table 1). It is to be noted that most of the co-infections were detected from the dead birds opportunistically sampled during the study. The presence of co-infections between different *Leucocytozoon* sp. lineages in the same individual were evidenced in some of the analyzed samples (Table 1). The high prevalence of co-infections was not a surprising result, since Paridae birds are known for having co-infections between different haemosporidian genera, and within the same parasite genus [41, 53].

The difficulties of morphological characterization of *Leucocytozoon* spp. have been discussed previously [50]. In the present study, we could not confirm the *Leucocytozoon* sp. involved in the infections as this requires us to investigate the type-host in the type locality, which has not yet been done. While the blood stages do not provide enough information to delimit *Leucocytozoon* species [50], underestimating parasite diversity, their *cytb* genetic diversity can overestimate its diversity [19, 22]. Nonetheless, including other morphological features, type of host cell infected by the parasite, parasite biology, and molecular information is essential to try to untangle the species characterization of *Leucocytozoon* spp.

Approximately 130 different lineages of *Leucocytozoon* spp. have been reported to infect Paridae birds, with 64 of them reported in Great tits, 33 in Blue tits, and 13 in Coal tits (MalAvi database, http://130.235.244.92/Malavi/, accessed on February 22, 2024). In the present study, we could confirm the presence of eight of them. Among the isolated *Leucocytozoon* spp. lineages, lPARUS4 was the most common one, present in five individuals (Table 1). This *Leucocytozoon* sp. lineage was previously reported to develop in lymphocytes and was associated with the *L. majoris* morphotype [8]. During this study, we could associate the lineages lSYBOR07 and lPARUS16 to the *L. majoris* morphotype; however, due to the absence of early stages of *Leucocytozoon* spp., we could not confirm on which type of host cell the parasite develops. The *Leucocytozoon* spp. lineages lPARUS4 and lPARUS19 have frequently been reported in the three bird species mentioned above [4]; in this study, the presence of both lineages was confirmed in two of the three species (Table 1).

No *Leucocytozoon* sp. infections were found in the analyzed Coal tits, which were infected only with *Plasmodium* sp. pBT7 (Table 1). This could be due to the low number of individuals analyzed in this study. Additionally, when compared to the other Paridae species, the prevalence of haemosporidians in Coal tits is lower. Numerous studies reporting new *Leucocytozoon* spp. lineages in wild birds are based only on molecular methodologies. Some authors point out that reports based on PCR alone may be the result of DNA amplification of sporozoites persisting in the bird after injection by the vector. However, some of these sporozoites may be unable to continue development in non-adapted hosts [13, 50], which does not allow us to know whether the infection is real or abortive [28, 34, 42, 44, 45]. Therefore, more studies targeting Coal tits should be encouraged to understand whether the prevalence of haemosporidian infections in this species is low.

Microscopic analysis of thin blood smears of birds infected with *Leucocytozoon* sp. lPARUS22 revealed the presence of *L. fringillinarum* morphotype (Figs. 1C–1D). The phylogenetic analyses placed this parasite lineage in a separate clade than the ones with *L. majoris* morphotype (Fig. 2). To date, associating the morphospecies *L. majoris* with its molecular information is challenging, resulting in difficulties in determining the phylogenetic relationships of the species within the phylogeny of *Leucocytozoon* spp. [8, 51]. It is noteworthy that *Leucocytozoon* sp. lPARUS22 clustered with other parasite lineages that have similar morphological features (*e.g.*, the nucleus of the infected cell assumes a cap-like form, which is one of the main features of *L. fringillinarum*) [21, 49]. *Leucocytozoon fringillinarum* was described in Eurasian chaffinch *Fringilla coelebs* in England [49], but to date, no *L. fringillinarum* lineage has been associated with the type species and type locality [50, 51]. Based on microscopic analyses, it was reported that parasites displaying the *L. fringillinarum* morphotype possibly develop in thrombocytes, supporting the importance of this character in phylogenetic relationships within *Leucocytozoon* spp. Two other *Leucocytozoon* lineages, lTFUS04 and lZOLEU02, have been pointed out as *L. fringillinarum*; however, they are placed in different and separated branches in the phylogenetic analysis (Fig. 2) [8, 44]. Based on morphological analyses, parasites with this morphotype have a wide distribution and the only zoogeographic region where *L. fringillinarum* has not been reported is Antarctica [49]. This may be because lZOLEU02 was isolated from a White-crowned sparrow *Zonotrichia leucophrys* and lTFSU04 from a Great thrush *Turdus fuscarter*, in the United States and Colombia, respectively. More studies using an integrative approach, including a combination of molecular and morphological techniques, are necessary to better understand the relationships within the genus *Leucocytozoon* and its relationship with other Haemosporida.

The genetic distance estimate between clades A and B, exceeding 7.9%, indicates that these two groups are likely distinct taxonomic entities. However, analysis of the genetic distances within each clade – 2.7% for clade A and 3% for clade B – suggests that the observed divergence may result from the clustering of lineages into multiple subclades within each group. This implies that the genetic diversity within each clade is higher than expected. Further investigation is required to explore this phenomenon in more detail.

It should be noted that none of the lineages obtained in this study have previously been associated with any described *Leucocytozoon* morphospecies. Additionally, several investigations reporting *Leucocytozoon* sp. infections are conducted mainly using molecular analysis [19]. The main reason for this is the lack of experts in parasitology for morphological investigations and species identification [5]. Morphological analysis of thin blood smears should be planned and encouraged in future studies, as well as training in species identification of avian blood parasites, since some characters can be of great value for better understanding phylogenetic analyses and for description of new parasites species.

Research focused on exo-erythrocytic stages has been carried out for some *Leucocytozoon* species, but these studies were mainly based on experimental infections [11, 14, 18, 26, 29–31, 36, 43, 56], with only a few reports on natural infections [1, 9, 27, 50]. The *Leucocytozoon* spp. life cycle begins when sporozoites are injected into the avian host by the infected Simuliidae insect during the blood meal, and these sporozoites can be persistent for up to 11 days, the longest persistence time of sporozoites in birds [14]. The role of this feature is still unknown and needs to be studied further [50]. After that, sporozoites invade the parenchymal cells in the liver, where the first generation of merogony occurs [49]. First-generation meronts produce individual merozoites and clusters of nuclei within the cytoplasm, known as “syncytia”. Subsequent generations of meronts, which stem from merozoites created by hepatic meronts, are able to mature within various fixed tissue cells, such as hepatocytes, renal epithelial cells, endothelial cells, and macrophages throughout multiple organs. These subsequent generations are especially common in the spleen and lymph nodes [50]. In the current study, it was not possible to confirm which generations of merogony were present in these infections. The development of meronts was evidenced in two individuals of Great tits (Figs. 3B–3F). The differences in the lengths of meronts found in both the liver (no larger than 30 μm) and the kidneys (no larger than 50 μm), and the high agglomeration of blood stages in capillary vessels suggest asynchronous development of these parasites in their avian hosts. It is also necessary to consider that the merogonial cycles of *Leucocytozoon* spp. can differ depending on the host species. In experimental infections, the number of generations of tissue stages of this genus is still unknown [51].

The complete exo-erythrocytic development of *L. majoris* has not been reported in the literature, and the tissue development of this parasite in their avian hosts is completely unknown. Although *Leucocytozoon* spp. parasite seems to be widely distributed in passerines, given the numerous lineages reported (MalAvi database, http://130.235.244.92/Malavi/ accessed on February 22, 2024), studies directed at understanding the merogonial development of these parasites need to be developed. However, it is known that some *Leucocytozoon* species, such as *L. fringillinarum,* can initiate merogony in the kidneys and liver [29, 30]. An experimental study investigated the exo-erythrocytic development of *L. fringillinarum* obtained from *Zonotrichia leucophrys*, and injected in *Quiscalus quiscula* [29, 30]. Meronts found in the kidneys were usually larger than those found in the liver, and they took longer to complete their development. However, in this experiment the authors reported that only the liver and kidneys were analyzed, so it is not possible to confirm whether the tested parasite can develop tissue stages in other organs [29, 30]. It is worth mentioning that the development of irregular meronts in the kidneys and the formation of cytomeres during merozoites development seems to be a general pattern in the maturation of *Leucocytozoon* spp. parasites [30, 50].

In our study, meront stages were found only in two individuals: H44/21R, with *Leucocytozoon* spp. unknown lineage (due to co-infection of lineages), and H58/21R *Leucocytozoon* sp. lPARUS16. The gametocytes found in both individuals were of *L. majoris* morphotype, and meronts were found only in the liver and kidneys. The megalomeront found in the heart of one analyzed Great tit (with lineage lPARUS22 which clusters with other lineages that resemble the *L. fringillinarum* morphotype) presented a markedly enlarged and deformed host cell nucleus and a thick capsule-like structure surrounding it (Figs. 3H, 3I). It can be inferred that this was a growing megalomeront, given the absence of individual distinguishable merozoites within the structure (Figs. 3H, 3I). The wall surrounding it is quite thick (≥ 1 μm), and deformed cells and fibers are present around it (Fig. 3I), which is part of the formation of the capsular wall [49, 51]. Furthermore, studies of other haemosporidian parasites of the genus *Haemoproteus* reported that the intensity of the signal produced by CISH may be different between mature (where the signal is weak) and growing (where the signal is more intense) megalomeronts. This could be because of the difference in ribosomal content of the mature stages of the parasite [16, 17, 24]. The same pattern could also be applied to the *Leucocytozoon* sp. megalomeront found in this study where an intense signal in the CISH-labelled structure was observed (Figs. 3H–3I). No meronts in any organ were found in this individual, suggesting that the infection by this parasite already had at least one generation of tissue stage, given that experimental infections have shown that the formation of megalomeronts is associated with the second or later generations of development in *Leucocytozoon* spp. [49–51]. This suggests that the stage found in this individual may be associated with a persistent stage of the parasite in the host, responsible for relapse of the infection. It is important to note that to have a more general overview of the parasite’s life cycle, we should have data from July to August, the period of active transmission at our study site [49]. Unfortunately, in our dataset, this period is not represented, which may or may not be related to the low prevalence of stages in organs. The developmental patterns of the exo-erythrocytic stages of *Leucocytozoon* spp. in most of the naturally infected passerines are still unknown, which shows that more research should be done in this field to better understand the presence of these stages. While general conclusions on the patterns of exo-erythrocytic development of *Leucocytozoon* spp. in Paridae birds cannot be drawn based on three positive individuals, the findings indicate possible directions for future investigations.

## Conclusion

*Leucocytozoon* spp. develop different types of exo-erythrocytic stages in natural infections in Great tits. *Leucocytozoon* sp. meronts were mainly found in the kidneys, although they can also be found in the liver, reinforcing the presence of asynchronous development. One growing megalomeront was found in the heart, confirmed by the CISH signal intensity, indicating that some *Leucocytozoon* sp. lineages can also infect other organs than those mentioned above, which could represent a serious threat to bird health. The development of certain parasite stages, such as megalomeronts, could be associated with the parasite lineage, as it was found in a single infection with a parasite resembling the morphospecies of *L. fringillinarum*. Co-infections (*Haemoproteus* sp.*, Plasmodium* sp., and *Leucocytozoon* sp.) were reported in several analyzed individuals. Parasites with morphological features resembling *L. majoris* and *L. fringillinarum* morphotypes clustered in different clades in the phylogenetic tree. Phylogenies based on partial *cytb* genes seem to indicate the morphology of parasite-host cell complexes in *Leucocytozoon* sp. Further studies should be carried out to understand the dynamics, pathogenicity, and life stages of *Leucocytozoon* spp. in wild birds.

## Supplementary material

The supplementary material for this article can be found at https://www.parasite-journal.org/10.1051/parasite/2025007/olm. *Supplementary Table S1*: GenBank accession numbers for the sequences used in the phylogenetic analysis.
